# Performance Evaluations of LoRa Wireless Communication in Building Environments

**DOI:** 10.3390/s20143828

**Published:** 2020-07-09

**Authors:** Ruobing Liang, Liang Zhao, Peng Wang

**Affiliations:** 1 Faculty of Infrastructure Engineering, Dalian University of Technology, Dalian 116024, China; liangrb@dlut.edu.cn (R.L.); ibewp@dlut.edu.cn (P.W.); 2 Key Laboratory of Intelligent Control and Optimization for Industrial Equipment of Ministry of Education, Dalian University of Technology, Dalian 116024, China; 3 School of Control Science and Engineering, Dalian University of Technology, Dalian 116024, China

**Keywords:** smart building, Internet of Things, LoRa technology, wireless communication

## Abstract

The Internet of things presents tremendous opportunities for the energy management and occupant comfort improvement in smart buildings by making data of environmental and equipment parameters more readily and continuously available. Long-range (LoRa) technology provides a comprehensive wireless solution for data acquisition and communication in smart buildings through its superior performance, such as the long-range transmission, low power consumption and strong penetration. Starting with two vital indicators (network transmission delay and packet loss rate), this study explored the coverage and transmission performances of LoRa in buildings in detail. We deployed three LoRa receiver nodes on the same floor and eight LoRa receiver nodes on different floors in a 16-story building, respectively, where data acquisition terminal was located in the center of the whole building. The communication performance of LoRa was evaluated by changing the send power, communication rate, payload length and position of the wireless module. In the current research, the metrics of LoRa were quantified to facilitate its practical application in smart buildings. To the best of our knowledge, this may be the first academic research evaluating RTT performance of LoRa via practical experiments.

## 1. Introduction

With the continuous progress of sensor and Internet of Things (IoT) technologies, increasing attention has been paid to the human comfort and building safety. On average, 80% of people’s time is spent in buildings, such as their homes, offices, schools, gymnasiums, etc. Therefore, new solutions are always expected to make buildings safer, more efficient, sustainable and comfortable [1,2]. Nowadays, smart building (SB) has attracted much concern, and it’s believed there will be increasing sensors and devices connected with each other and further intelligently used in the field of smart buildings in the near future.

SB is an attractive topic for researchers due to its ability of real-time dynamic control over different activities and energy consumption reduction in building operations [3]. The wireless communication technology, sensors and IoT technology are often used by SB to (1) transmit and analyze data and (2) further control and optimizing building management systems. The combination of IoT solutions is used for automated access control, security systems, lighting, HVAC (heating, ventilation and air conditioning) systems [4,5]. Besides the achieved goals of owners, managers and tenants, they are also able to provide a greater efficiency, safety and comfort, with much less costs. For example, in the office of SB, the environment and energy consumption could be automatically configured and effectively managed by monitoring the air conditioning, lighting, computers, windows, doors and other components, especially the human behaviors [6]. In SB, the indoor environment parameters and energy consumption can be detected in real time by installing wireless sensors, while the collected data can be adjusted automatically to guarantee the most comfortable environment, both of which are able to reduce energy consumption and improve resource utilization efficiency. SB has become a system engineering based on the information collection, intelligent control, IoT, data analysis and management. The use of IoT will significantly improve the overall performance of SB and achieve a high integration and intelligent application. The development of SB will be accompanied by the development of IoT technology [5].

Data acquisition and communication are the foundation of SB which could be divided into wired and wireless communications. For the former one, it includes the Ethernet communication, BACnet communication, RS485 communication, power line carrier communication, etc. [7]. However, the high labor cost and difficult construction of wired communication hinder the development of SB. In recent years, with the rapid development of wireless communication technologies in the IoT, such as Zigbee, Wi-Fi, Bluetooth, LoRa, etc. [8], have been widely used in intelligent buildings. The use of wireless network is easy to expand, which reduces the difficulty of wiring construction and improves the system flexibility.

Table 1 compares the performances of several wireless communication modes, which may be suitable for the application in SB. It is clearly shown that compared with other three wireless communication technologies, the LoRa technology exhibits more advantages in the transmission distance and low energy consumption. However, due to the potential complex environment and the large loss of wireless signals in the building, LoRa technology will also face many problems.

The application of wireless network in building mainly includes the indoor environmental quality monitoring and indoor positioning. Firdhous et al. [9] proposed a Zigbee-based O_3_ concentrations monitoring system based on Bluetooth in offices by the photocopier machine. Liang Z et al. [10] designed an innovative indoor air quality detector with multiple communication interfaces, included LoRa, Wi-Fi GPRS and NB-IoT. The multiple interfaces made the proposed system possess a good compatibility and can be used on many occasions. In study [11], Chao-Tung Y proposed an intelligent indoor environment monitoring system (iDEMS) combined with ZigBee wireless sensor network technology, which could monitor temperature, humidity, CO, CO_2_ and VOC. A modular IoT platform based Zigbee was implemented in [12] for real-time indoor air quality monitoring, where authors gave a full description about hardware and software design, and the abilities of the system were also demonstrated by the collected results in various locations.

On the other hand, the research on indoor positioning technologies has been conducted for more than two decades. Yik [13], presented a decentralized BLE-based positioning protocol that did not require training before deployment. The training process could be done on the fly by anchor nodes automatically, with an accuracy of approximately 1.5 m. Andres et al. [14] proposed and evaluated a positioning system based on LoRa technology with RSSI indicator. Extensive measurements showed that position estimation errors were less than around 7% between LoRa modules. In [15], a Wi-Fi-based indoor localization was designed by using fuzzy classifier and multi-layer perceptron ensemble. The high accuracy and low mean error made it possible to apply Wi-Fi-based localization in real indoor parking lots.

The main research purpose of this study is to investigate the communication performance of LoRa wireless technology inside an office building. The transmission delay and packet loss rate of LoRa between the same floor and different floors were obtained by experimental measurements. Then the main influencing factors of LoRa wireless communication were analyzed to provide basic data support and engineering reference for the application of LoRa technology in intelligent buildings. The innovation and contributions of this study are listed follows:

- The penetration performance of LoRa on the same floor and different floors are studied with the transmitter deployed in the central position of the whole building;
- The effects of payload lengths on the LoRa RTT (round-trip time) delay and packet delivery rate are investigated;
- The effects of air rates on the LoRa RTT delay and packet delivery rate are discussed;
- The effects of communication power on LoRa RTT delay and packet delivery rate are explored;
- The effects of different distances and locations in buildings on LoRa RTT delay and packet delivery rate are studied.

The remainder of this study is organized as follows. The related studies about smart building and LoRa technology are introduced in Section 2. The measurement setup is presented in Section 3 in detail, which discusses test scenarios, LoRa module and distribution of data acquisition terminal and data response terminals, which are acted by air quality detectors (AQDs) with LoRa wireless module in them. Section 4 analyzes experimental results and Section 5 provides the discussion. Finally, this study is concluded in Section 6.

## 2. Background and Related Works

### 2.1. Smart Building

**Figure 1:**
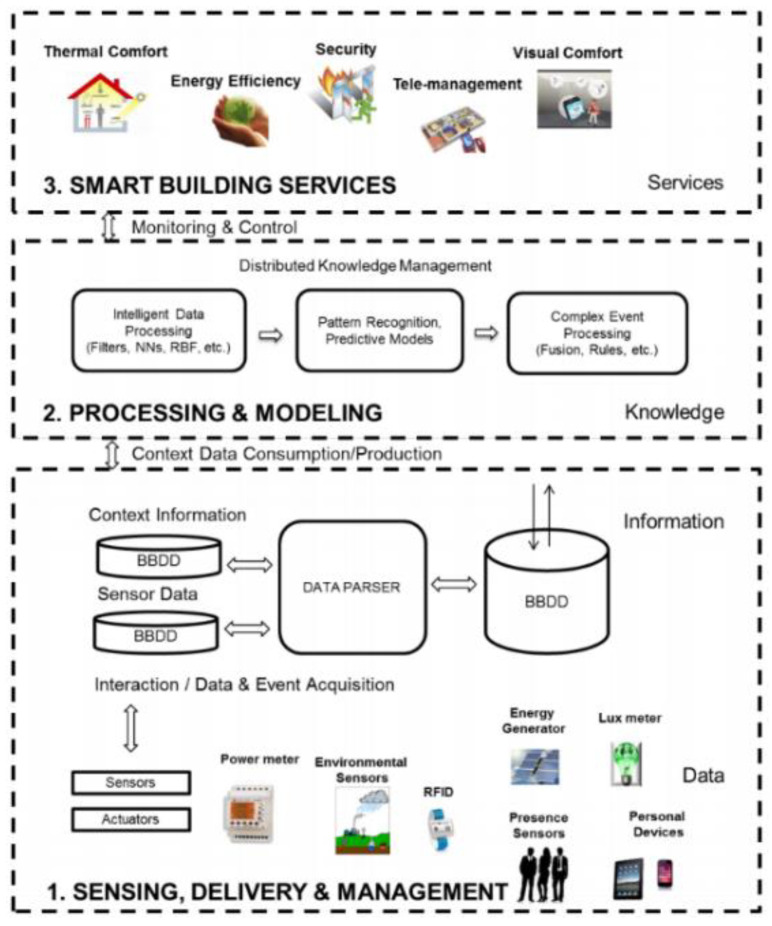
Conceptual layered approach for smart buildings [17].

SB plays an important field of automatic treatment and control of temperature, humidity, ventilation, safety, lighting and other parameters during the building operations. In addition, it plays a vital role in implementing the standards of improving enhanced living environment (ELE) and ambient assisted living (AAL) [16]. Figure 1 shows the three main conceptual layers of SB in an IoT-enabled environment, which consists of the information layer, responding for sensing, delivery and management layer, knowledge layer, in charge of processing and modeling, services layer, offering smart building services.

### 2.2. LoRa Technology

As one of IoT communication technologies, LoRa is a kind of ultra-long distance wireless transmission technology based on the spread spectrum transmission techniques and CSS (chirp spread spectrum) modulation, which is one of the IoT communication technologies. Its name comes from the abbreviation of “long range”. It can be seen from the name, the biggest feature of LoRa is the long distance communication. LoRa’s spread spectrum technology changes the balance between transmission power consumption and transmission distance, and completely changes the situation in the field of embedded wireless communication. This technology presents a new communication technology that enables long-distance, long battery life, large system capacity and low hardware costs to meet the IoT needs [18].

**Figure 2:**
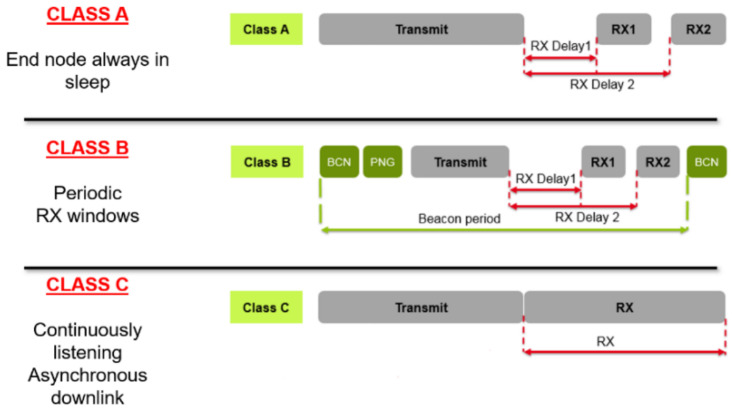
Three terminal operation modes of Lora [19].

According to the different application situations, the terminal equipment is divided into three different classes: Class A, Class B and Class C, as shown in Figure 2.

Class A: The uplink transmission of each terminal device will be accompanied by two downlink receiving windows. After the terminal sending an uplink transmission signal, the server can communicate with the downlink very quickly. At any time, the downlink communication of the server can only be later than the uplink one, which consumes the lowest power.

Class B: It owns a synchronous slot beacon and a fixed period receiving window ping slot, to receive data at intervals, while, at other times, they are sleeping. This method consumes the lowest power and least delay in downloading data from the server. At the same time, it helps the server get the information that whether the terminal device is receiving data, which is suitable for locators, switches and other scenarios.

Class C: This kind of terminal equipment keeps the receiving window open and only will be closed when the data are transmitted. Therefore, it consumes more power compared with Class A and Class B.

### 2.3. The Application and Performance of LoRa in Buildings

Numerous researches have studied the application of LoRa technology in SB under different scenarios, such as the safety monitoring [20], healthcare [21,22,23], power consumption monitoring [24], environmental quality monitoring [25], smart metering [26] and indoor personnel positioning [27].

In [20], a wearable LoRa based wireless sensor network was proposed to monitor the harmful environmental conditions, which adopted some self-powered environmental sensors. Data could be displayed to users through web-based applications on cloud servers. An IoT-based health monitoring system via LoRa was reported in [23] to collect physiological data such as blood pressure, glucose, and temperature from people in rural areas are transmitted to a remote LoRa server using the LoRaWAN network. In [18], a low-power real-time air quality monitoring system was proposed in building based on the LoRa to collect several air pollution parameters, such as NO_2_, CO, PM10 and PM2.5. A smart metering as developed based on the LoRa technology, which was applied to smart buildings and smart grid systems [26]. In [27], an indoor positioning technology was presented based on the RSSI intensity of LoRa. The measurement accuracy was further improved by ANN training and the positioning accuracy can reach 95%.

In addition to its application in each single building, LoRa technology was also widely used in complex buildings [22,24,28,29,30,31]. Guillermo et al. [24] proposed a power distribution monitoring for suburban area by using the LoRa technology and estimated LoRa coverage by simulation tools. In [22], Petajajarvi et al. presented a health and wellbeing monitoring system for whole campus area (570 m × 320 m). Their results showed that LoRa can achieve perfect coverage in the entire buildings in the campus, with the antenna mounted at a height of 24 m above the sea level. Wang et al. [31] have built an environment conditions monitoring in an university and deployed several LoRa nodes indoors and outdoors. The measured data showed that the packet loss rate in the outdoor under line-of-sight environment was much smaller than that in the indoor environment. In [29], Haxhibeqiri et al. discussed the performance of indoor LoRa in an industrial environment with 250,000 m^2^ cover area. The RSSI (received signal strength indication), SNR (signal noise ratio) and PLR (packet loss ratio) parameters of LoRa were evaluated in detail, and the measurement results showed that the total coverage area of LoRa would be larger with an increase of spreading factor (SF).

Several parameters of LoRa PHY (physical) can be adjusted to achieve different transmission performance, such as the SF, send power level, bandwidth, air rate and payload length. There have been numerous literatures focusing on the indoor coverage and transmission performance of LoRa in recent years. In [32], a detailed research of the radio propagation models and transmission performance of LoRa is presented in Lebanon. A series of experiments were carried out in both indoor and outdoor environment at rural and urban areas. In order to achieve a good transmission effect, increasing the number of gateways was essential. Neumann et al. [33], analyzed the influence of position change on the throughput, RSSI, SNR and PER (packet error ratio). It was found that the transmission signal was attenuated seriously in the basement and the wall of the ground part had little effect on the transmission signal. In [34], SF, CR (coding rate), PL (payload length) were selected to assess the RSSI and PER of LoRa by using SX1276 at 868 MHz. The results suggested that the value of SF should be changed for modules in different locations in order to obtain the optimal communication effect. Hosseinzadeh [35] and Gregora [36], researched the effects of distance and location distribution on RSSI, respectively. In [37], as a unique research, the LoRa propagation characteristics for both the 434 MHz and 868 MHz ISM bands were discussed. The measurement confirmed that, the 434 MHz had a superiority performance compared to 868 MHz.

Most of the previous literatures analyzed the LoRa performance in buildings based on the RSSI and PDR parameters. For end-users, besides the PDR (packet delivery ratio), time delay is also an important parameter that deserves close attention. To the best of our knowledge, there are no literatures systematically studied the impact of network delay on the LoRa performance, expert [38], which only analyzed the payload length on network time delay by simulation method. In order to guarantee the real-time performance of the control system in SB, it is often necessary to improve the transmission rate, which will lead to the decline of PDR. Therefore, how to balance the relationship between these parameters is a problem that must be faced in the application of SB.

## 3. Measurement Setup

### 3.1. Test Scenario & Node Placement

**Figure 3:**
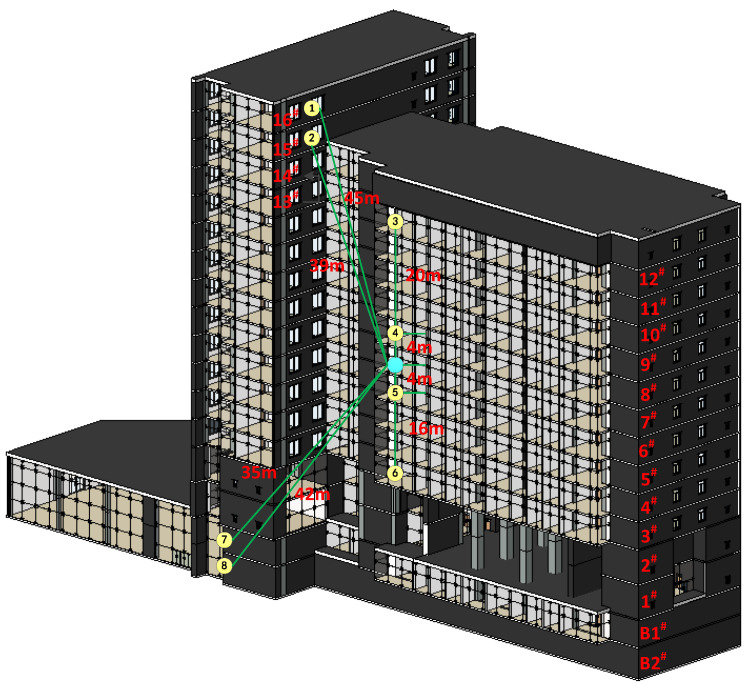
Model diagram of the measured building.

**Figure 4:**
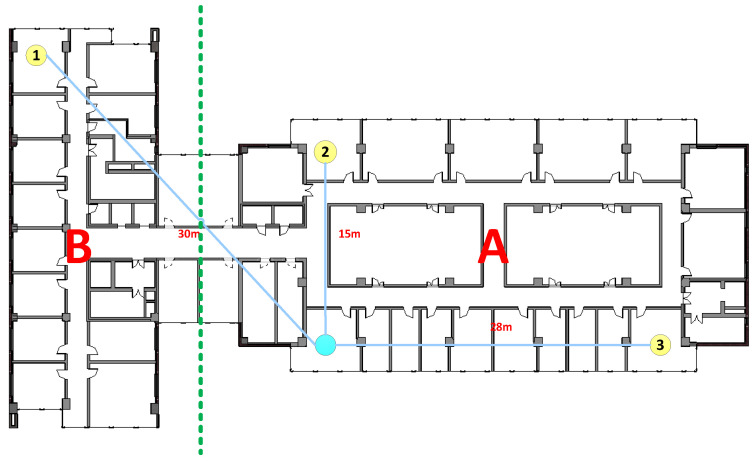
Floor-level detail of measured building.

The performance of LoRa was tested in a reinforced concrete office building, located in Dalian University of Technology in China as shown in Figure 3. The whole building is divided into two districts—A and B, of which 12 floors above the ground in area A, 16 floors above area B and 2 floors below ground. The first and second layers are five-meter-high, and the third and higher floors are four-meter-high, with a total height of 85.6 m. Figure 4 shows the detailed distribution from the 1st floor to the 12th floor.

As shown in Figure 3, the transmit LoRa module is located in the middle of the whole building, on 7th floor and in the horizontal position, the placement is also close to the center as shown in Figure 4. On the 7th floor, we place three receive LoRa modules located in the easternmost, westernmost and northernmost directions, respectively. The main purpose of doing so to measure the penetration performance of LoRa module on the cement wall and the effect of different distances on transmission delay. In order to measure LoRa’s penetration performance of reinforced concrete, we deployed eight receiving modules on different floors, as displayed in Figure 3.

### 3.2. LoRa Modules and Test Method

**Figure 5:**
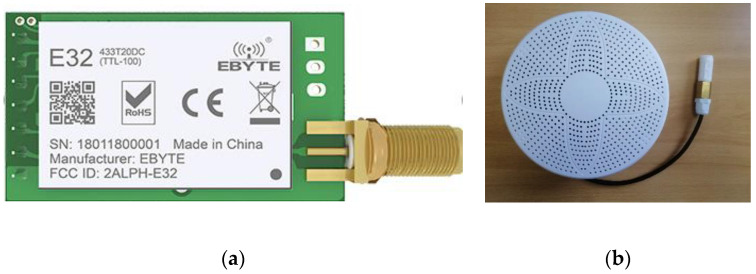
LoRa modules: (**a**) transmitter; (**b**) receiver.

The basic design of LoRa wireless network module is shown in Figure 5. It is based on a Semtech SX1278 LoRa RF 433 MHz transmitter with maximum transmit power 20 dBm, and the maximum communication distance can reach 3000 m at the line of sight condition. The receiver is an air quality detector (AQD) with LoRa communication [10]. The basic parameters of LoRa module are listed as in Table 2.

**Figure 6:**
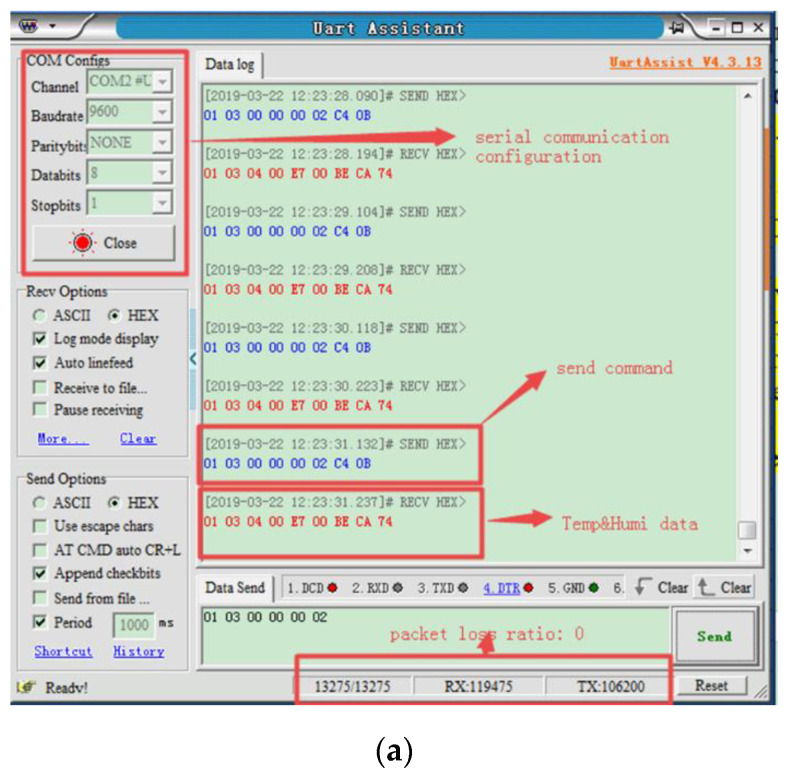

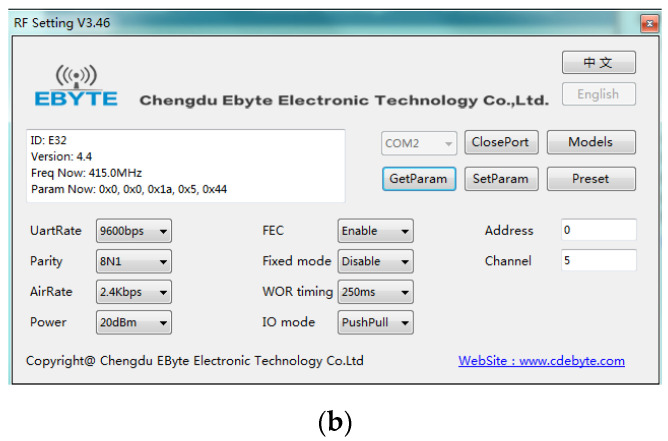
Software used in test: (**a**) measurement software [39]; (**b**) configuration software [40].

The procedure of the test is designed as follows: (1) first, a collect command is sent in the format of Modbus protocol through LoRa transmitter on the 7th floor by using Uart assistant software, as shown in Figure 6a and (2) then all the AQDs within the communication distance will receive this command, but only one AQD matching address will feedback a message to transmitter. The configuration software is shown in Figure 6b which is used to verify the performances of LoRa module under different transmit powers and air rates.

### 3.3. Reliability Metrics

There are many reliability indicators to evaluate the performance of wireless network, such as the RSSI, SNR, PDR, PER, LQI (link quality indication), transmission time delay et al. Most of the indicators in the previous literatures were focused on the RSSI, SNR, PDR [29,30,31,34,35,36,37]. However, for end-users, they do not care about RSSI and SNRs of wireless signals. From qualitative sense, the wireless network reliability means that the desired data are sent to the receiver at the desired times, with least time delay and minimal PDR [41]. Hence, this study focuses on these two indicators to evaluate the network performance of LoRa inside the building scenario.

#### 3.3.1. Round-Trip Time (RTT)

**Figure 7:**
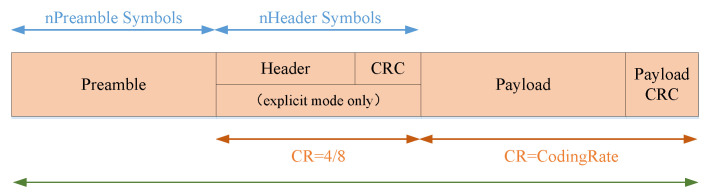
Packet structure of LoRa.

Figure 7 shows the packet structure of LoRa, which consists of preamble, header, CRC (cyclic redundancy check), payload and payload CRC. The TOA (time-on-air) can be calculated by Equation (1).

(1) $$ToA=T_{s}*(T_{pre}+\max(\Lambda *(CR+r),0))$$

(2) $$\Lambda =\frac{8PL-4SF+28+16CRC-20H}{4*(SF-2DE)}$$

(3) $$T_{s}=\frac{2^{SF}}{BW},\;T_{pre}=(n_{pre}+12.25)$$

By obtaining the for spreading factor (SF), coding rate (CR) and signal bandwidth (BW), the total transmission time for a single LoRa packet can be calculated using the above formulas.

In the end-to-end time delay measurement of network communication, the common method is to get the end-to-end delay value directly by sending the probe package. If the transmission delay of LoRa has to be estimated by Equation (1), the calculation process is too complex. On the other hand, RTT is generally used instead of end-to-end time delay to evaluate the performance. In general, RTT is the time that the sender experiences from the time moment when the data are sent to the moment when it receives feedback packet from the receiver. The measurement software is used to record the sending and receiving time points and the RTT can be obtained by receiving time points minus sending time points.

#### 3.3.2. Packet Delivery Rate (PDR)

PDR is the ratio of packets successfully received to the total sent. In this test, the PDR is calculated by using measurement software, as it could record total sent and received packets, as shown in Figure 6a. By changing the acquisition instructions of Modbus protocol, the return payload PL of AQD can be adjusted to evaluate the effect of communication messages of different length on PDR. Furthermore, SP (send power) and AR (air rate) of LoRa module can be changed by configuration software shown as in Figure 6b to verify the performance of LoRa module under different SPs and ARs.

## 4. Experimental Results and Analysis

In this section, we describe the experimental results and analysis. The experimental measurement is divided into three groups: the first one is the RTT measurement experiment, the second and the third ones are the PDR measurement experiment. Three AQDs are located on the 7th floor in the second group, to evaluate the penetration performance of the LoRa module on the cement wall. Eight AQDs are deployed on different floors to evaluate LoRa’s penetration of reinforced concrete. In order to ensure the accuracy of the test results, the following assumptions were made the present study:

(1) The performances of all LoRa wireless modules used in the test are consistent with each other.
(2) When testing on the same floor, the wall thickness of each room does not change.
(3) When testing on different floors, the floor thickness between floors is the same.
(4) The processing delay of data acquisition terminal and AQDs is constant.

The parameter settings for the three sets of experiments are depicted in Table 3.

### 4.1. RTT Measurement Experiments

In the RTT experiment, we change the transmitted power, payload length, air rate and position of receivers. As shown in Table 3, only one parameter was changed in each set of experiments, leaving the remaining parameters unchanged, and each set of tests measured ten sets of RTT data.

#### 4.1.1. RTT vs. Transmitted Power

**Figure 8:**
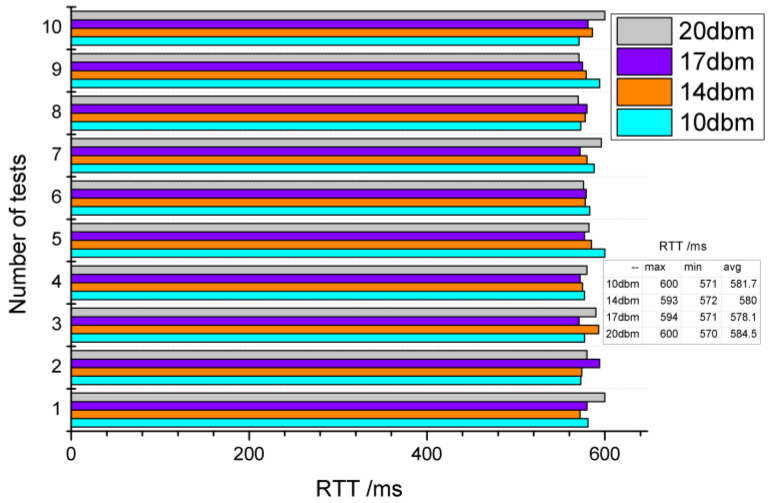
Relationship between round-trip time (RTT) and send power.

This experiment tested the RTT of two nodes under different transmitted power, including SP = 10 dBm, 14 dBm, 17 dBm and 20 dBm. Figure 8 shows the relationship between RTT and SP. The maximum, minimum, and average of RTTs under SP = 10 dBm are 600 ms, 571 ms and 581.7 ms, respectively. While the maximum, minimum and average of RTTs under SP = 20 dBm are 600 ms, 570 ms and 584.7 ms, respectively. This experiment illustrates that RTT does not decrease as the SP increases, and different SP shows little effect on RTT.

#### 4.1.2. RTT vs. Payload Length

**Figure 9:**
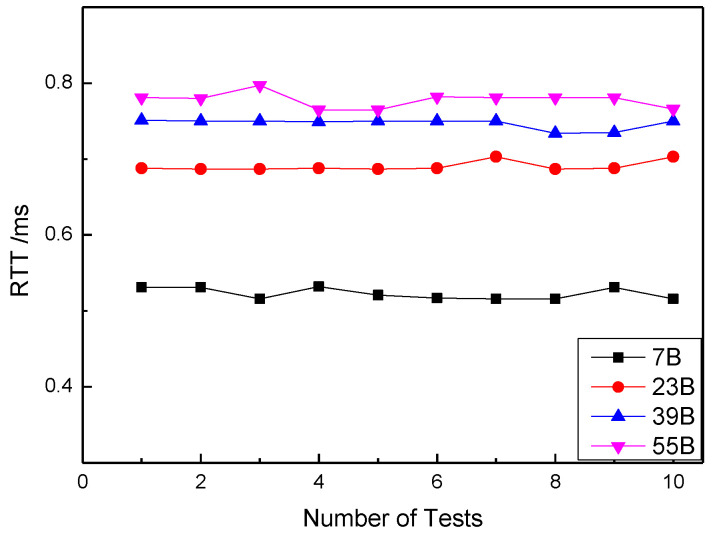
Relationship between RTT and payload length.

This experiment tested the RTT of two nodes under different payload length settings. Considering that the use of LoRa wireless communication in the building is mainly for data acquisition and control instruction transmission, the amount of data transmitted will not be too large, and four different payload lengths are designed here. Namely, PL = 7 B, 23 B, 39 B and 55 B. Figure 9 describes the relationship between RTT and PL. It can be clearly seen that the fluctuation of RTT is very small when the length of PL is fixed, and RTT increases with the increase of PL. However, when the packet size is larger than 23 bytes, the trend of delay increase slows down, and there are three points very close to each other under PL = 39 B and PL = 55 B. The length of each packet should be reasonably set to obtain the optimal data transmission results, and in some scenarios this may result in high real-time requirements.

#### 4.1.3. RTT vs. Air Rate

According to the design of the LoRa PHY layer, larger air rate should result in lower RTT. This experiment was conducted in order to investigate the relationship between RTT and AR.

**Figure 10:**
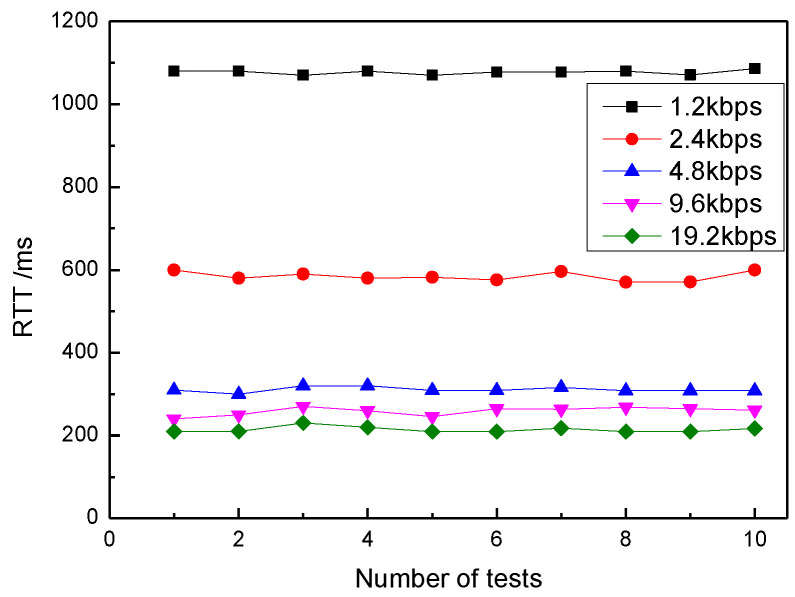
Relationship between RTT and air rate.

The setting are as follows: AR = 1.2 kbps, AR = 2.4 kbps, AR = 4.8 kbps, AR = 9.6 kbps and AR = 19.2 kbps. Figure 10 illustrates how RTT was affected by different AR settings under PL = 7 B and SP = 20 dBm. The data showed that with the increase of AR, RTT decreases rapidly. When AR is larger than 9.6 kbps, the downward trend of transmission delay becomes slower. When the AR value is constant, the RTT of each group of tests changes very little, indicating that the transmission path is stable. The increase of AR can reduce RTT, but it will also lead to communication instability. We will discuss the impact of different AR on packet loss rate in the following experiments.

#### 4.1.4. RTT vs. Location

**Figure 11:**
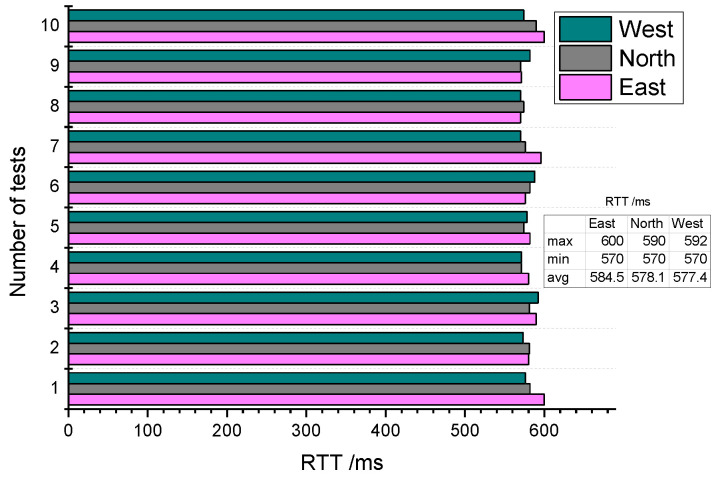
Relationship between RTT and position.

This experiment tested the relationship between RTT and different locations. There are three AQDs deployed on the 7th floor, in the northernmost, easternmost and westernmost directions. There are two cement walls and two wooden doors between the transmitter and the northernmost AQD. There are nine cement walls between transmitter and the easternmost AQD, and the distance is nearly 28 m, the AQD on the west side is located about 30 m away from the transmitter, and the blocking situation in the middle is complex. Figure 11 shows how RTT changes with different locations. Similar to the first SP change test, the RTT values of these three positions did not fluctuate significantly.

Statistical data showed that the minimum RTT of the three locations is 570 ms, and the maximum value is 590 ms in the north, 592 ms in the west and 600 ms in the east. This is consistent with the actual distribution complexity and the distance from the transmitter point, but the difference between these values is not very obvious. On the other hand, the minimum average occurs in the westernmost AQD, which also shows that there is no significant difference in RTT between the three locations.

### 4.2. PDR Measurement on the Same Floor

The purpose of the PDR experiments was to measure the penetration performance of the wireless signal of LoRa inside the building. In this section, we deployed three AQDs on the 7th floor, distributed in the easternmost, northernmost and westernmost, respectively. As with the same configuration of previous experiments, only one parameter was changed in each set of experiment, leaving the remaining parameters unchanged. The transmitter was set to collect data at 10 s intervals for more than one and a half hours, and the transmitter can send more than 500 acquisition packets. PDR can be calculated by dividing the number of packets returned by that of sent.

**Figure 12:**
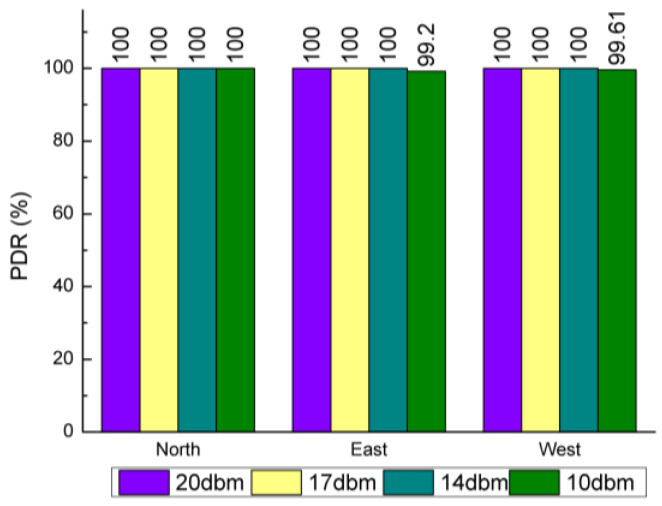
Relationship between packet delivery rate (PDR) and send power in the same floor.

**Figure 13:**
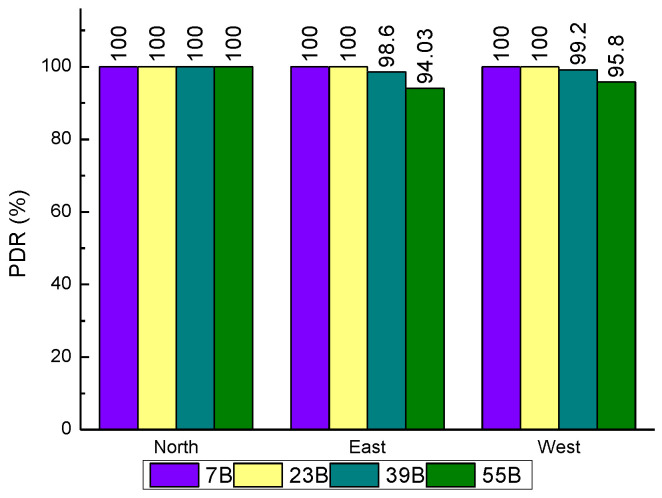
Relationship between PDR and payload length in the same floor.

**Figure 14:**
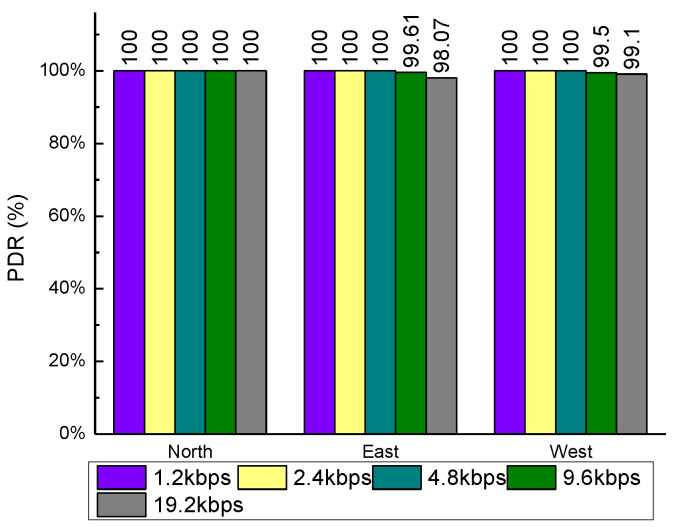
Relationship between PDR and air rate in the same floor.

Figure 12, Figure 13 and Figure 14 showed the plots of the PDR under three scenarios where the northernmost point yielded the best PDR. The PDRs measured were 100% no matter how the parameters changed. When the power was reduced to 10 dBm, the PDRs in the easternmost and westernmost measuring points showed a small decrease, but the magnitude of the decrease was very small, less than 1 percentage point. When the air rate was higher than 9.6 kbps, the PDRs measured in the easternmost and westernmost both began to decline to a small extent, with the largest decrease occurring in the easternmost under 19.2 kbps condition, but still less than 2 percentage points. Compared with the above two scenarios, changing the size of the data packet shows a relatively greater impact on the PDR. When the data packet reached 55 B, the packet loss rate dropped to 94.03%, and the lowest PDR still appeared at the easternmost measurement point. Overall, the LoRa wireless module performance on the same floor is excellent, with the average PDR measurement exceeding 99.5%. The measurement results showed that LoRa can obtain high PDR even though it needs to penetrate nine cement walls.

### 4.3. PDR Measurements on Different Floors

In this part, PDR is measured on different floors, and the parameters are set exactly in accordance with our previous experiment.

#### 4.3.1. PDR vs. Transmitting Power

**Figure 15:**
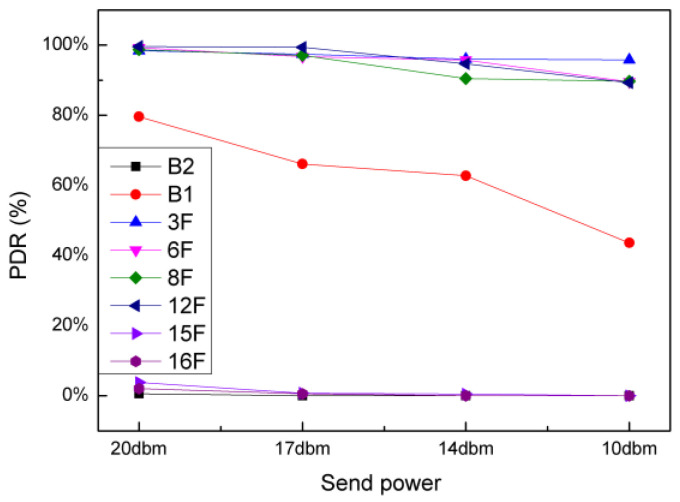
Relationship between PDR and send power in the different floors.

This experiment tested the PDR of two nodes under different send power settings, including SP = 10 dBm, 14 dBm, 17 dBm and 20 dBm. Figure 15 shows the relationship between PDR and SPs on the different floors. It can be clearly observed that the PDR of several locations far from the launcher is very low (B2, 15 F, 16 F, we called it far area), close to 0. On the other hand, the position closer to the launcher obtains high PDRs (3 F, 6 F, 8 F, 12 F, we called it near area). However, with the gradual reduction of SP, PDR shows a significant downward trend, but the decline is slow. It is noteworthy that the PDR at B1 position has its own characteristics. The measurement results of B1 position fluctuate greatly, but the overall trend is still downward.

#### 4.3.2. PDR vs. Payload Length

**Figure 16:**
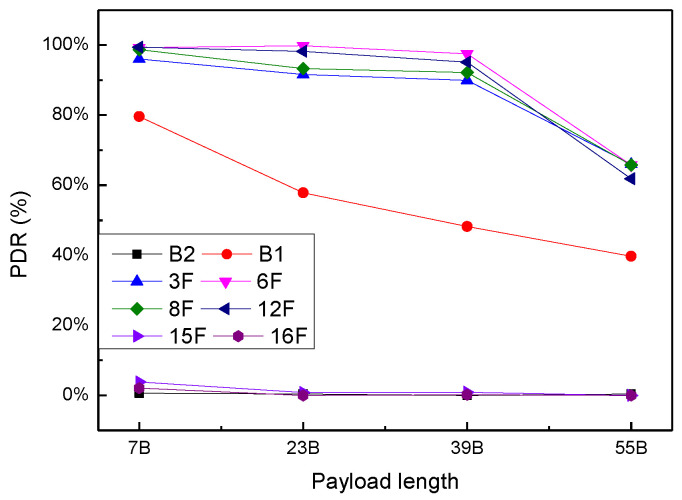
Relationship between PDR and payload length in the different floors.

This experiment tested the PDR of two nodes under different payload length settings, including PL = 7 B, 23 B, 39 B and 55 B. Figure 16 describes the relationship between PDR and PLs on the different floors. In the near area, with the increase of PL, PDR shows a downward trend, but when the data length is less than 39 B, the downward speed is very slow, and when the data length reaches 55 B, the PDR decreases significantly. The PDR of 55 B is 30% lower than that of 39 B. On the other hand, in the far area, the measurement results are not satisfactory, and the PDR is still close to 0. For B1 point, PDR decreases stepwise with the increase of PL. The PDR of 55 B is 40% lower than that of 7 B.

#### 4.3.3. PDR vs. Air Rate

**Figure 17:**
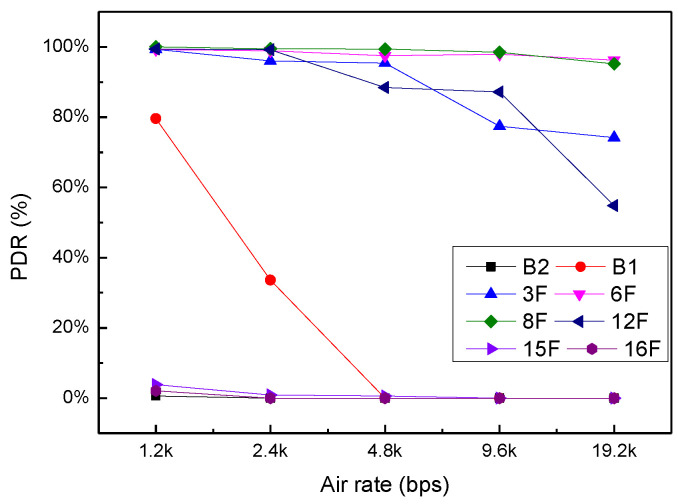
Relationship between PDR and air rate in the different floors.

This experiment tested the PDR of two nodes under different air rate settings, including AR = 1.2 kbps, AR = 2.4 kbps, AR = 4.8 kbps, AR = 9.6 kbps and AR = 19.2 kbps. Figure 17 illustrates the relationship between PDR and ARs on the different floors. Similar to the two experiments conducted above, the PDRs in the far zone are still close to 0. The PDRs in the near area decrease with the increase of ARs, but the downward trend of 6 F and 8 F is more moderate, while the downward trend of 3 F and 12 F is more obvious. When AR = 19.2 kbps, the PDR for 3 F and 12 F decreases by 21.5% and 40.9%, respectively, compared with 6 F and 8 F.

#### 4.3.4. PDR vs. Position

**Figure 18:**
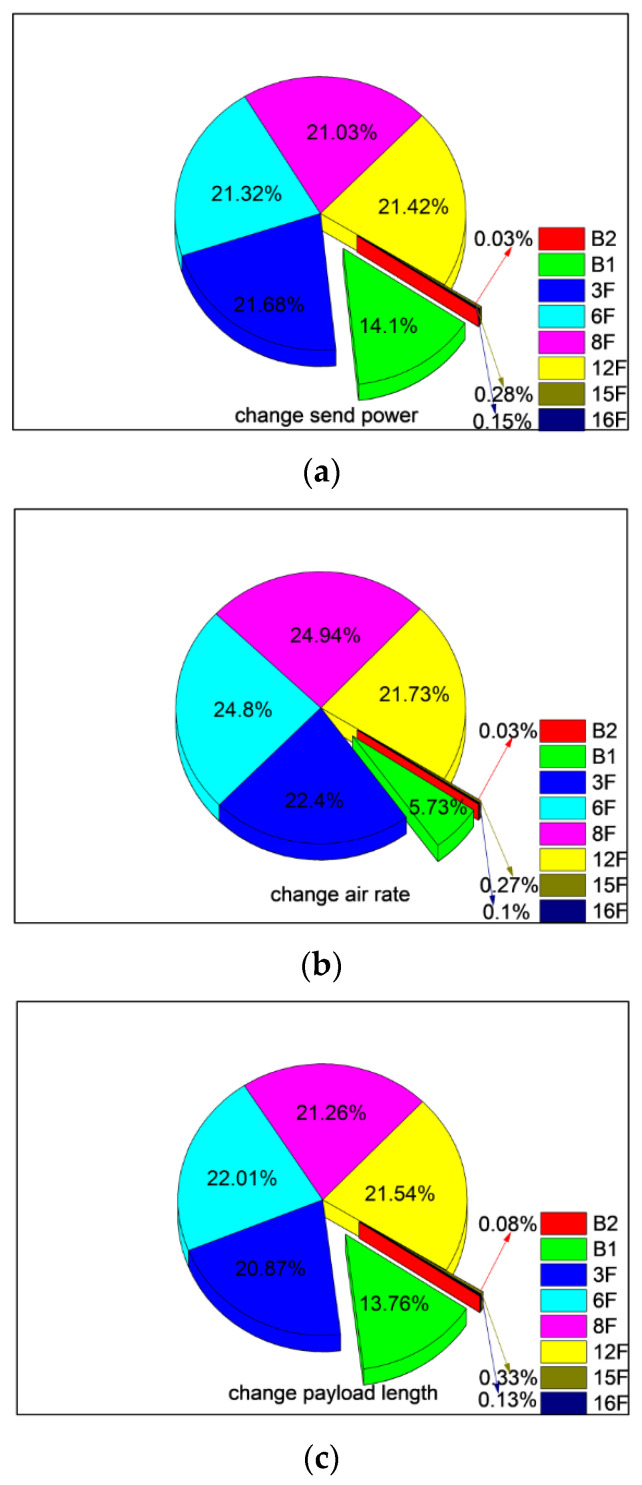
Relationship between PDR and position in different floors. (**a**) Change send power; (**b**) Change air rate; (**c**) change air rate.

In order to investigate the communication performance of LoRa wireless module with the change of different floors, we counted the PDR data of above three groups of experiments and analyzed the proportion of PDR in each experiment scenarios. The statistical results are shown as in Figure 18. The percentages of PDR in the near area are 85.4%, 85.7%, 93.9%, respectively, which correspond to SP, PL and AR changing experiment scenario, respectively. On the other hand, the corresponding PDR of far area is only 0.4%, 0.5% and 0.4%, respectively. As for B1, AR has the greatest impact, the smallest percentage of PDR occurred in the AR change group, which was only 5.73%. When SP and PL changed, the percentage of PDR of B1 reached 14.1% and 13.76%, respectively.

## 5. Discussion

This study focused on the data transmission performance of LoRa in a building without considering its power consumption. During the experiments, the collector was wall powered and the AQDs were powered by portable power source with 10,000 mAh, which had the ability to support AQD for 12 consecutive hours. Based on the above experiments and results, we can draw the following conclusions:

- Compared with transmitting power and position of LoRa module, AR and PL had more influence on RTT. When AR and PL were constant, the RTT fluctuated in the range of 570–600 ms by changing the position and SP;
- RTT increased with the increase of PL and decreased with the increase of AR. However, when the PL was larger than (23 B)—or AR was larger than (9.6 kbps)—the change trend of RTT became moderate;
- It could be seen from the comparative test of the same floor and different floors that the PDR of the same floor was always greater than 95%. The PDRs in the far zone (B2, 15 F, 16 F) were close to 0 and even in the near zone (3F, 6F, 8F, 12F), there were still many PDRs less than 80%. Compared with reinforced concrete, LoRa had better penetration performance for the cement wall;
- On the whole, PDR increased with the increase of send power and decreased with an increase of payload and air rate. Furthermore, payload length and air rate had greater influence than transmitting power;
- For the location selection of the collector, it may be a better choice to place it in the center of the whole building than on the roof.

In the process of the third group of experiments, there is one detail that needs to be paid attention to, namely point B1. Although B1 and B2 were located very close, the measurement data of these two points were quite different. The reason for this may origin from the building structure. The position from the first to the third floor directly in Area A was hollow, which was just below the transmitter and reduces the transmission resistance of wireless signals. Hence, the data at point B1 are inconsistent with those at point B2.

Table 4 presents a comprehensive and comparative analysis of proposed performance evaluations of LoRa in building environments with various literatures in recent five years. Most of the previous literatures focused on the 868 MHz, and only two papers researched 434 MHz [29]. However, the penetration performance of 868 MHz is worse than 434 MHz, which is not suitable for building applications in theoretical. In addition, previous literatures mostly researched RSSI, SNR and PDR indicators, there are few academic studies to research on time delay or RTT of LoRa.

## 6. Conclusions

The long-distance transmission of LoRa wireless technology makes it possible to be widely used in smart buildings, but the practical concerns of signal reliability in different building environments still lead to reluctance in practical use. In this study, two vital metrics, RTT and PDR, are considered for use in evaluating the reliability of LoRa signal propagation. RTT is an important parameter to measure the real-time performance of control system, and PDR can quantify the reliability of wireless communication system effectively.

From the end-user’s point of view, this study focused on the indoor propagation of LoRa signal and the main contribution of this work is to measure the RTT and PDR by changing send power, payload length and air rate in a multi-level building. The measurement was conducted in a reinforced concrete building, where a total of ten groups of experiments were conducted to assess the performance of LoRa by designing three scenarios. The research results of this study are helpful to the practical application of LoRa wireless communication technology in smart buildings. Further work will focus on the performance under mesh networking condition, such as Zigbee or Wi-Fi in response to a larger building complex system. On the other hand, we also consider designing some low-power wireless network communication systems to meet the inconvenient power supply scenarios.

## Figures and Tables

**Table 1:** Comparison of typical wireless communication technologies [8].

**Parameters**	**Wi-Fi**	**LR-WPAN**	**Bluetooth**	**LoRa**
Standard	IEEE 802.11 a/c/b/d/g/*n*	IEEE 802.15.4 (Zigbee)	IEEE 802.15.1	LoRaWAN R1.0
Frequency band	5–60 GHz	868/915 MHz, 2.4 GHz	2.4 GHz	868/900 MHz
Data rate	1–6.75 Gb/s	40–250 Kb/s	1–24 Mb/s	0.3–50 Kb/s
Transmission range	20–100 m	10–20 m	8–10 m	<30 km
Energy consumption	High	Low	Bluetooth: Medium BLE: Very low	Very low

**Table 2:** The parameters of LoRa module.

Band (MHz)	Send Power (dBm)	Receive Sensitivity (dBm)	Air Rate (kbps)	Work Current (mA)
410–441	10–20	−130	1.2–19.2	102-send 20 dBm 90-send 10 dBm 12-receive

**Table 3:** Parameters setting of the experiment.

Test	SP (dBm)	PL (Bytes)	AR (bps)	Position
A.1	change	7	2400	line of sight
A.2	20	change	2400	line of sight
A.3	20	7	change	line of sigh
A.4	20	7	2400	change
B.1	change	7	1200	7th floor
B.2	20	change	1200	7th floor
B.3	20	7	change	7th floor
C.1	change	7	1200	B2-16th
C.2	20	change	1200	B2-16th
C.3	20	7	change	B2-16th

**Table 4:** Comparative analysis of literature.

Ref.	Year	ISM Band	LoRa Module	Reliability Metrics	Parameters
[27]	2017	868 MHz	SX1276	RSSI, PER	SF, CR,
[22]	2017	912 MHz	NA	PLR	Payload, antenna angle, distance, weather
[28]	2017	868 MHz	SX1272	RSSI	Distance
[30]	2016	NA	SX1301	RSSI	location
[23]	2018	430 MHz	SX1278	PDR	SF, location, hop, SNR
[29]	2018	434 MHz, 868 MHz	RN2483	SNR	ISM band
[24]	2017	868 MHz	iC880A	RSSI, SNR, PLR	Location
[26]	2016	868 MHz	IC880A	Throughput, RSSI, SNR, PLR	Location
This study	2020	433 MHz	SX1278	PLR, RTT	Location, payload, power, CR

## Nomenclature

AAL	Ambient assisted living
ANN	Artificial neural network
AQD	Air quality detector
AR	Air rate
BW	Bandwidth
CR	Coding rate
CRC	Cyclic redundancy check
CSS	Chirp spread spectrum
ELE	Enhanced living environments
IoT	Internet of things
ISM	Industrial scientific medical
LQI	Link quality indication
PDR	Packet delivery ratio
PER	Packet error ratio
PHY	Physical
PL	Payload length
PLR	Packet loss ratio
RS	Receive sensitivity
RSSI	Received signal strength indication
RTT	Round-trip time
SB	Smart building
SF	Spreading factor
SP	Send power
SNR	Signal noise ratio
TOA	Time of arrival

## References

[1] Tran T.V., Dang N.T., Chung W.Y. (2017). Battery-free smart-sensor system for real-time indoor air quality monitoring. Sens. Actuators B Chem..

[2] Bhattacharya S., Sridevi S., Pitchiah R. Indoor air quality monitoring using wireless sensor network. Proceedings of the 2012 Sixth International Conference on Sensing Technology.

[3] Zainordin N.B., Abdullah S.M.B., Baharum Z.B.A. (2012). Light and Space: Users Perception towards Energy Efficient Buildings. Procedia-Soc. Behav. Sci..

[4] Bo W.H., Zhang Y.L., Sun H.R., Huang X. Usable Security Mechanisms in Smart Building. Proceedings of the IEEE 17th International Conference on Computational Science and Engineering (CSE).

[5] Mussab A., Zaidan A.A., Mohammed T., Kiah M.L.M. (2017). A review of smart home applications based on Internet of Things. J. Netw. Comput. Appl..

[6] Sembroiz D., Careglio D., Ricciardi S., Fiore U. (2019). Planning and operational energy optimization solutions for smart buildings. Inf. Sci..

[7] Zhao L., Zhang J.L., Liang R.B. (2013). Development of an energy monitoring system for large public buildings. Energy Build..

[8] Ray P.P. (2018). A Survey on Internet of Things Architectures. J. King Saud Univ.-Comput. Inf. Sci..

[9] Firdhous M., Sudantha B., Karunaratne P. IoT enabled proactive indoor air quality monitoring system for sustainable health management. Proceedings of the 2nd IEEE International Conference on Computing and Communications Technologies (ICCCT).

[10] Zhao L., Wu W.Y., Li S.M. (2019). Design and implementation of an IoT based indoor air quality detector with multiple communication interfaces. IEEE Internet Things J..

[11] Yang C.T., Chen S.T., Den W., Wang Y.T., Kristiani E. (2019). Implementation of an Intelligent Indoor Environmental Monitoring and management system in cloud. Future Gener. Comput. Syst..

[12] Mohieddine B., Abderrazak A., Sabbir A., Farid T., Abdullah K. (2018). A Modular IoT Platform for Real-Time Indoor Air Quality Monitoring. Sensors.

[13] Ho Y.H., Chan H.C.B. (2020). Decentralized adaptive indoor positioning protocol using Bluetooth Low Energy. Comput. Commun..

[14] Andres V.R., Fabian A.S., Christian S., Braulio A., Luis I.M. (2020). Experimental evaluation of RSSI-based positioning system with low-cost LoRa devices. Ad Hoc Netw..

[15] Zhu Q.W., Xiong Q.Y., Wang K., Lu W., Liu T. (2019). Accurate WiFi-based indoor localization by using fuzzy classifier and mlps ensemble in complex environment. J. Frankl. Inst..

[16] Younus M.U., Islam S.U., Ali I., Khan S., Khan M.K. (2019). A survey on software defined networking enabled smart buildings: Architecture, challenges and use cases. J. Netw. Comput. Appl..

[17] Hernández-Ramos J.L., Moreno M.V., Bernabé J.B., Carrillo D.G., Skarmeta A.F. (2015). SAFIR: Secure access framework for IoT-enabled services on smart buildings. J. Comput. Syst. Sci..

[18] Zhang X.H., Zhang M.M., Meng F.F., Qiao Y., Xu S.J., Senghout H. (2019). A Low-Power Wide-Area Network Information Monitoring System by Combining NB-IoT and LoRa. IEEE Internet Things J..

[19] Polonelli T., Brunelli D., Marzocchi A., Benini L. (2019). Slotted ALOHA on LoRaWAN-Design, Analysis, and Deployment. Sensors.

[20] Wu F., Redoute J.M., Yuce M.R. (2018). WE-Safe: A Self-Powered Wearable IoT Sensor Network for Safety Applications Based on LoRa. IEEE Access.

[21] Wu F., Wu T.Y., Rasit Y.M. (2019). An Internet-of-Things (IoT) Network System for Connected Safety and Health Monitoring Applications. Sensors.

[22] Juha P., Konstantin M., Rumana Y., Matti H., Jari L. (2017). Evaluation of LoRa LPWAN Technology for Indoor Remote Health and Wellbeing Monitoring. Int. J. Wirel. Inf. Netw..

[23] Mdhaffar A., Chaari T., Larbi K., Jmaiel M., Freisleben B. IoT-based health monitoring via LoRaWAN. Proceedings of the IEEE EUROCON 17th International Conference on Smart Technologies.

[24] Campo G.D., Gomez I., Calatrava S., Martinez R., Santamaria A. Power Distribution Monitoring Using LoRa: Coverage Analysis in Suburban Areas. Proceedings of the 2018 International Conference on Embedded Wireless Systems and Networks.

[25] Liu S., Xia C., Zhao Z. A low-power real-time air quality monitoring system using LPWAN based on LoRa. Proceedings of the 13th IEEE International Conference on Solid-State and Integrated-Circuit Technology (ICSICT).

[26] Abdelfatteh H., Abdelhak A., Aziz D. Performance Evaluation of Low-Power Wide Area based on LoRa Technology for Smart Metering. Proceedings of the 6th International Conference on Wireless Networks and Mobile Communications.

[27] Muekdang S., San-Um W. (2018). Intelligent RF-Based Indoor Localization through RSSI of LoRa Communication Technology. IEEE Access.

[28] Catherwood P.A., David S., Mike L., Stephen M., James L. (2018). Community-Based IoT Personalized Wireless Healthcare Solution Trial. IEEE J. Transl. Eng. Health Med..

[29] Haxhibeqiri J., Karaagac A., Van den Abeele F., Joseph W., Moerman I., Hoebeke J. LoRa indoor coverage and performance in an industrial environment: Case study. Proceedings of the IEEE International Conference on Emerging Technologies and Factory Automation-ETFA.

[30] Lee H., Ke K. (2018). Monitoring of Large-Area IoT Sensors Using a LoRa Wireless Mesh Network System: Design and Evaluation. IEEE Trans. Instrum. Meas..

[31] Wang S.Y., Chen Y.R., Chen T.Y., Chang C.H., Cheng Y.H., Hsu C.C., Lin Y.B. Performance of LoRa-Based IoT Applications on Campus. Proceedings of the IEEE Vehicular Technology Conference.

[32] Rida E.C., Samer L., Melhem E.H. (2019). LoRaWAN Network: Radio Propagation Models and Performance Evaluation in Various Environments in Lebanon. IEEE Internet Things J..

[33] Neumann P., Montavont J., Noel T. Indoor deployment of low-power wide area networks (LPWAN): A LoRaWAN case study. Proceedings of the IEEE International Conference on Wireless & Mobile Computing.

[34] Ayele E.D., Hakkenberg C., Meijers J.P., Zhang K., Meratnia N., Havinga P.J.M. Performance analysis of LoRa radio for an indoor IoT applications. Proceedings of the 2017 International Conference on Internet of Things for the Global Community (IoTGC).

[35] Salaheddin H., Hadi L., Krystyna C., Andrew W., Amin A. Empirical propagation performance evaluation of LoRa for indoor environment. Proceedings of the IEEE International Conference on Industrial Informatics INDIN.

[36] Lukas G., Lukas V., Marek N. Indoor signal propagation of LoRa technology. Proceedings of the International Conference on Mechatronics-Mechatronika.

[37] Thomas A., Patrick V.T., Hendrik R. LoRa Indoor Performance: An Office Environment Case Study. Proceedings of the 2018 International Applied Computational Electromagnetics Society Symposium-China (ACES).

[38] Xia Z., Zhou H., Yin B., Zeng Y., Xu M. (2018). Secure Session Key Management Scheme for Meter-reading System Based on LoRa Technology. IEEE Access.

[39] Measurement Software of Experiment. https://download.cnet.com/Uart-Assist-for-Windows-10/3000-2352_4-77561194.html.

[40] Configuration Software of Experiment. http://www.ebyte.com/pdf-down.aspx?id=664.

[41] Jang W.S., Healy W.M. (2010). Wireless sensor network performance metrics for building applications. Energy Build..

